# Feasibility of endoscopic hand suturing on rectal anastomoses in ex vivo porcine models

**DOI:** 10.1038/s41598-021-01396-y

**Published:** 2021-11-08

**Authors:** Eriko Koizumi, Osamu Goto, Seiichi Shinji, Koki Hayashi, Tsugumi Habu, Kumiko Kirita, Hiroto Noda, Kazutoshi Higuchi, Takeshi Onda, Jun Omori, Teppei Akimoto, Mitsuru Kaise, Hiroshi Yoshida, Katsuhiko Iwakiri

**Affiliations:** 1 Department of Gastroenterology, Nippon Medical School Graduate School of Medicine, 1-1-5 Sendagi, Bunkyo-ku, Tokyo, 113-8603 Japan; 2 Department of Gastrointestinal and Hepato-Biliary-Pancreatic Surgery, Nippon Medical School, Tokyo, Japan

**Keywords:** Cancer, Gastroenterology

## Abstract

Prevention of postoperative anastomotic leakage in rectal surgery is still required. This study investigated the feasibility of endoscopic hand suturing (EHS) on rectal anastomosis ex vivo. By using isolated porcine colon, we prepared ten anastomoses 6–10 cm from the virtual anus. Then, we sutured anastomoses intraluminally by EHS, which involved a continuous suturing method in 5 cases and a nodule suturing method with extra corporeal ligation in 5 cases. Completeness of suturing, number of stitches, procedure time and presence of stenosis were investigated. Furthermore, the degree of stenosis was compared between the two suturing methods. In all cases, EHS were successfully completed. The median number of stitches and procedure time was 8 and 5.8 min, respectively. Stenosis was created in all continuous suturing cases whereas none was seen in nodule suturing cases. The shortening rate was significantly greater in the continuous suturing method than in the nodule suturing method. Intraluminal reinforcement of rectal anastomosis by EHS using nodule suturing with extra corporeal ligation is feasible without stenosis, which may be helpful as a countermeasure against possible postoperative anastomotic leakage in rectal surgery.

## Introduction

Anastomotic leakage is one of the most serious complications of lower anterior resection for rectal cancer. That may cause perioperative death and poor prognosis^1,2^, and is also considered to be a major influential factor on organ/space surgical site infection that occurs in approximately 5% of colorectal surgery^3^. In addition, it sometimes requires a permanent stoma, which remarkably worsens the patients’ quality of life. The reported incidence of anastomotic leakage after rectal cancer resection ranges from 3 to 27%^4–6^, and several risk factors including male sex, history of ischemic heart disease and distal anastomotic location are proposed^5,6^.

Recently, the double-stapled anastomotic technique has been widely used due to its advantage to create anastomosis at a lower position in the pelvis than before without losing the sphincter function^7,8^. However, this method cannot avoid making a “dog ear,” an ischemic area surrounded by some staple lines, which can lead to anastomotic leakage postoperatively^9^. Although several reinforcing methods to prevent leakage after mechanical anastomosis are proposed so far^4,9,10^, effective countermeasures to reduce the risk of anastomotic leak sufficiently have not been established.

We developed endoscopic hand suturing (EHS), which is a novel endoscopic suturing technique that allows optimal and secure intraruminal suturing with a curved needle and surgical suture. This technique has been introduced clinically, and is expected for preventing postoperative bleeding after endoscopic submucosal dissection by closing mucosal defects^11–13^. We hypothesized that application of EHS for anastomosis to reinforce the anastomosis trans-anally may be useful for reduction of the risk of anastomotic leakage. In this study, we investigated the feasibility of EHS on sites of mechanical anastomosis in lower anterior resection in an ex vivo porcine model. In addition, we also compared the degree of stenosis in the two suturing techniques (continuous suturing and nodule suturing).

## Materials and methods

### Feasibility of endoscopic hand suturing on anastomoses

#### Preparation

**Figure 1:**
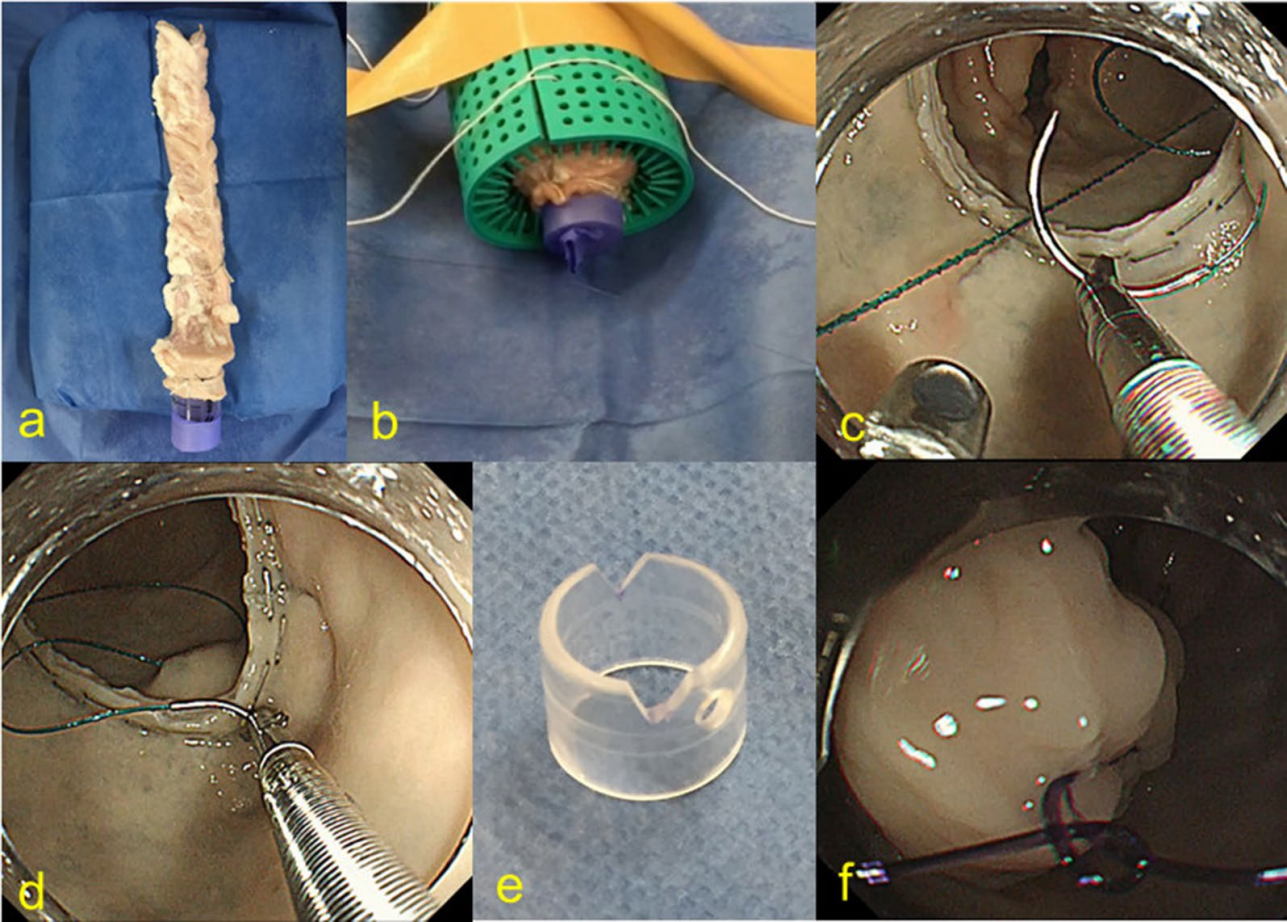
Endoscopic hand suturing for rectal anastomosis. (**a**) A porcine rectal model with anastomosis which is made with double stapling technique. (**b**) The rectal model having a virtual anus is fixed on a rubber roll with string. (**c, d**) The full layer at the proximal side of the anastomosis is sutured using a flexible needle holder (**c**), followed by suturing at the distal side (**d**). (**e**) In nodule suturing, a notched transparent straight hood is used. (**f**) A extracorporeally-created knot is delivered to the anastomotic site using the endoscope.

We prepared ten portions of the colon, 20 cm in length, isolated from pigs that had been previously slaughtered for food. After rinsing with tap water, each colon was cut in two and closed one edge with a surgical suturing instrument (ENDO GIA ULTRA SHORT; Covidien Japan Corp., Tokyo, Japan: ENDOGIA TRISTAPLE 60 AMT; Covidien Japan). Then, anastomosis was created 6–10 cm from another edge with the ENDO GIA and a surgical anastomosis instrument (EEA 28MM SINGLE-USE STAPLER; Covidien Japan). We manually created a virtual anus with a holed figure part of a surgical globe mounted onto a 3 cm- cut syringe, and fixed them on a rubber roll with string (Fig. 1a, b).

### Endoscopic hand suturing

All procedure was conducted using a multi-bending scope with two working channels (GIF-2TQ260M; Olympus Co. Ltd., Tokyo, Japan). A transparent straight hood (D-201-13, 404; Olympus) was attached to a tip of the endoscope. The suturing procedure described below was conducted by one endoscopist and one assistant who had had clinical experience in EHS.

**Figure 2:**
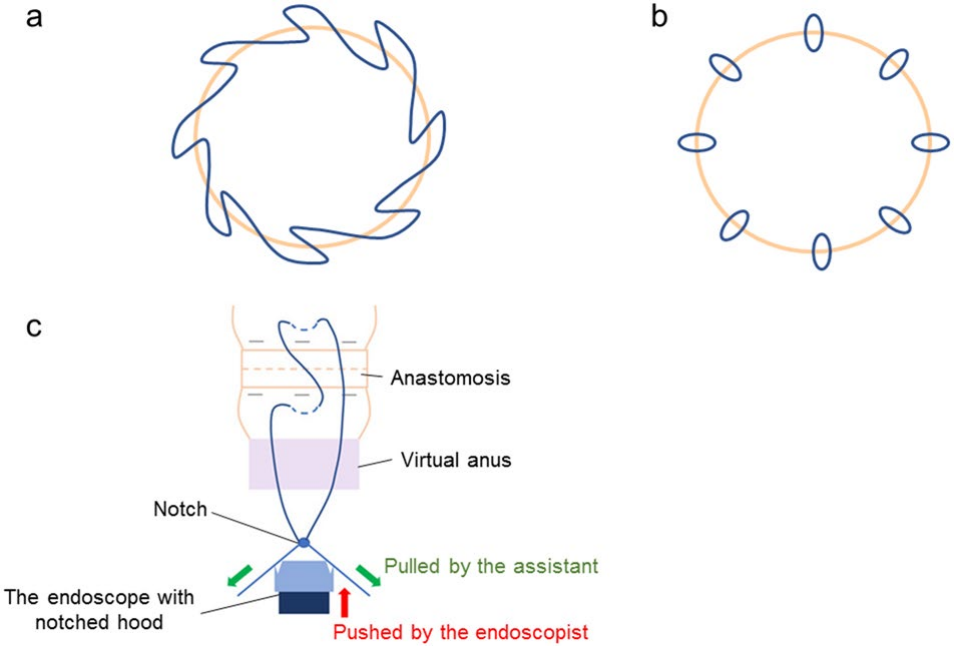
Schemas of endoscopic hand suturing for rectal anastomosis. (**a**) The continuous suturing with a barbed suture. The anastomosis was sutured circumferentially. (**b**) The nodule-suturing method. (**c**) In the nodule suturing, the slipknot is created outside the tract after retrieving the needle. The knot is delivered to the lumen by using the tip of the endoscope with the notched hood (red direction) while the thread remains tensioned by the assistant (green direction).

In five cases, we performed EHS with a continuous suturing method in the same manner as previously introduced^13^. As the first step of EHS, a 3–0 V-loc 180™ absorbable barbed suture (VLOCL0604; Covidien, Mansfield, Massachusetts, USA), which had short barbs to prevent the suture from sliding backward after the tissues being tightened, was delivered through the virtual anus by using the prototype of the flexible needle holder (Olympus) by grasping the thread close to the tail of the needle. The suture thread was once released and the body of the needle was grasped. Then, the needle was passed through the full layer on the proximal part of the anastomosis, followed by passing through the layer on the distal side (Fig. 1c, d). We circumferentially sutured the layers between the anastomosis at intervals of a little less than 1 cm. The remaining portion of the thread was cut with the dedicated scissors forceps (Olympus) and transanally retrieved by grasping the thread part (Fig. 2a).

In other five cases, we performed EHS with a nodule suturing method with extra corporeal ligation. In these cases, the transparent hood attached to the tip of the endoscope had been notched (about 3 mm) at 3 and 9 o’ clock in advance (Fig. 1e). The suture thread used was a 3–0 absorbable monofilament suture (Biosyn™; Covidien Japan). The needle was delivered and passed through both sides of anastomosis in the same way as continuous suturing cases. Subsequently, the needle was completely retrieved outside. The endoscopist tied the thread with slipknot and hooked each side of the thread to corresponding notches at 3 and 9 o’clock in the hood. Then, while the assistant was pulling the thread, the endoscopist delivered the knot using the endoscope itself as a knot pusher to the anastomotic site (Fig. 1f). We carried this procedure four times per stitch and cut the remaining thread with scissors forceps. Finally, the needle was retrieved by pulling the thread. The intervals of stitches were set at approximately 1 cm (Fig. 2b, c).

### Comparison of degree of stenosis between two suturing methods

After rinsing the isolated colon with tap water, the tract was cut longitudinally and separated every 10 cm. Accordingly, a total of ten colonic models in approximately 10 cm squares were prepared and pinned to the urethan boards. An endoscopist manually sutured the mucosa in line eight times. In five models, we sutured with continuous suturing method by using 3–0 V-loc 180™, and other five models were sutured with nodule suturing method by using 3–0 Biosyn™.

### Outcome measures

In the experiment on feasibility, we assessed the completeness of the suturing, the number of stitches, average procedure time of suturing and presence of stenosis after suturing. Changes in procedure time of suturing in the nodule suturing cases were also investigated. The procedure time of suturing was defined as the duration between the first insertion of the needle tip and the final cut of the remaining thread in each case. In the experiment on degree of stenosis, we compared an average of shortening rate of flat colon models between continuous suturing method and nodule suturing method.

### Statistical analysis

Obtained results are presented as median and range. For comparison of shortening rate between two suturing methods, t-test was used. *P* values < 0.05 were considered to indicate statistical significances. Statistical analyses were performed using IBM SPSS version 25 (IBM Corp., Armonk, NY, USA).

## Results

**Figure 3:**
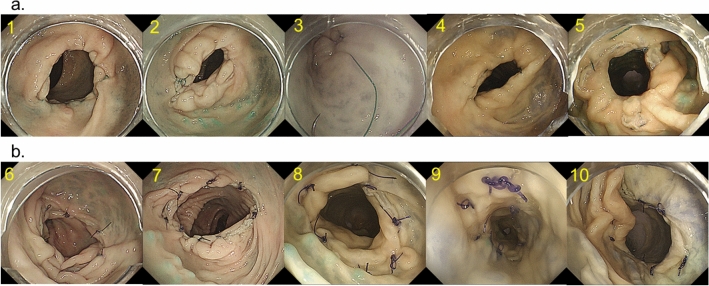
Endoscopic images of anastomosis before and after endoscopic hand suturing. (**a**) Cases sutured by the continuous suturing method (Case 1–5). Case 1, mild stenosis; Case 2, moderate stenosis; Case 3, severe stenosis; Case 4, moderate stenosis; Case 5, mild stenosis. (**b**) Cases sutured by the nodule suturing method (Case 6–10). No stenosis.

**Figure 4:**
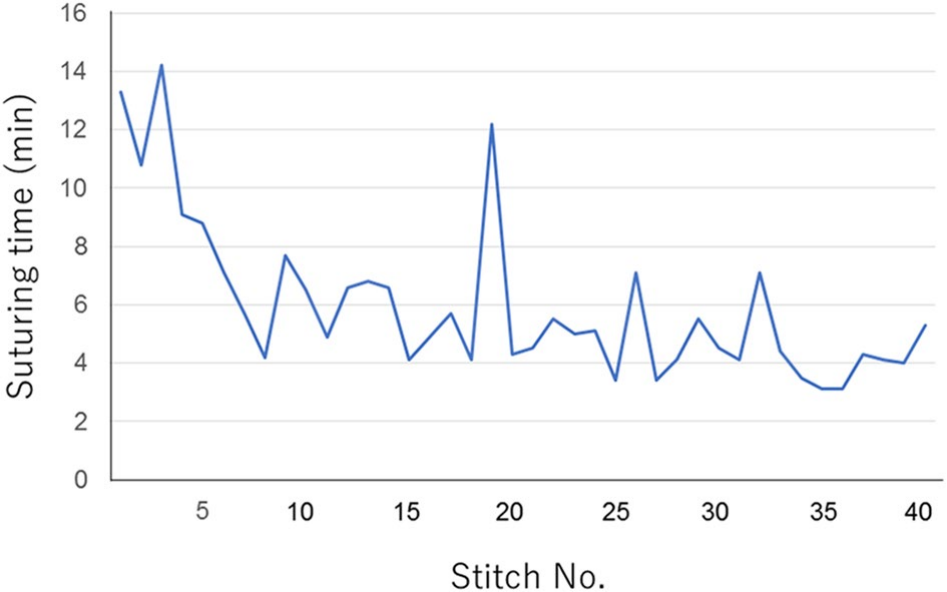
Changes in suturing time in the nodule-suturing method. The suturing time, defined as the duration required for creating a single nodule, becomes shorter as the number of stitches is accumulated.

In the experiment on feasibility of EHS with rectal anastomosis model, the EHS procedure was completed in all cases. The median number of stitches was 8 (range 6–9), and the median procedure time of suturing was 5.8 min (range 3.6–9.1 min). In the five cases with the continuous suturing method, stenosis was revealed (the first case, mild; the second case, moderate; the third case, severe, the fourth case, moderate; the fifth case, mild). In contrast, no stenoses were seen in the five cases in the nodule suturing method with extra corporeal ligation (Table 1, Fig. 3). The procedure time of suturing in the nodule suturing cases became shorter as the procedure proceeded (Fig. 4).

**Table 1:** Outcomes of endoscopic hand-suturing of rectal anastomosis in an ex vivo porcine model.

Case no.	Suturing method	Thread	Complete	Stitches, n	Median procesdure time of suturing, minutes	Stenosis
1	Continuous suturing	3-0 V-loc 180^TM^	Yes	9	5.6	Mild
2	Continuous suturing	3-0 V-loc 180^TM^	Yes	8	4.9	Moderate
3	Continuous suturing	3-0 V-loc 180^TM^	Yes	6	8.2	Severe
4	Continuous suturing	3-0 V-loc 180^TM^	Yes	8	3.3	Moderate
5	Continuous suturing	3-0 V-loc 180^TM^	Yes	8	3.6	Mild
6	Nodule suturing	3-0 Biosyn^TM^	Yes	8	9.1	No
7	Nodule suturing	3-0 Biosyn^TM^	Yes	8	6.0	No
8	Nodule suturing	3-0 Biosyn^TM^	Yes	8	7.3	No
9	Nodule suturing	3-0 Biosyn^TM^	Yes	8	6.6	No
10	Nodule suturing	3-0 Biosyn^TM^	Yes	8	5.4	No

**Figure 5:**
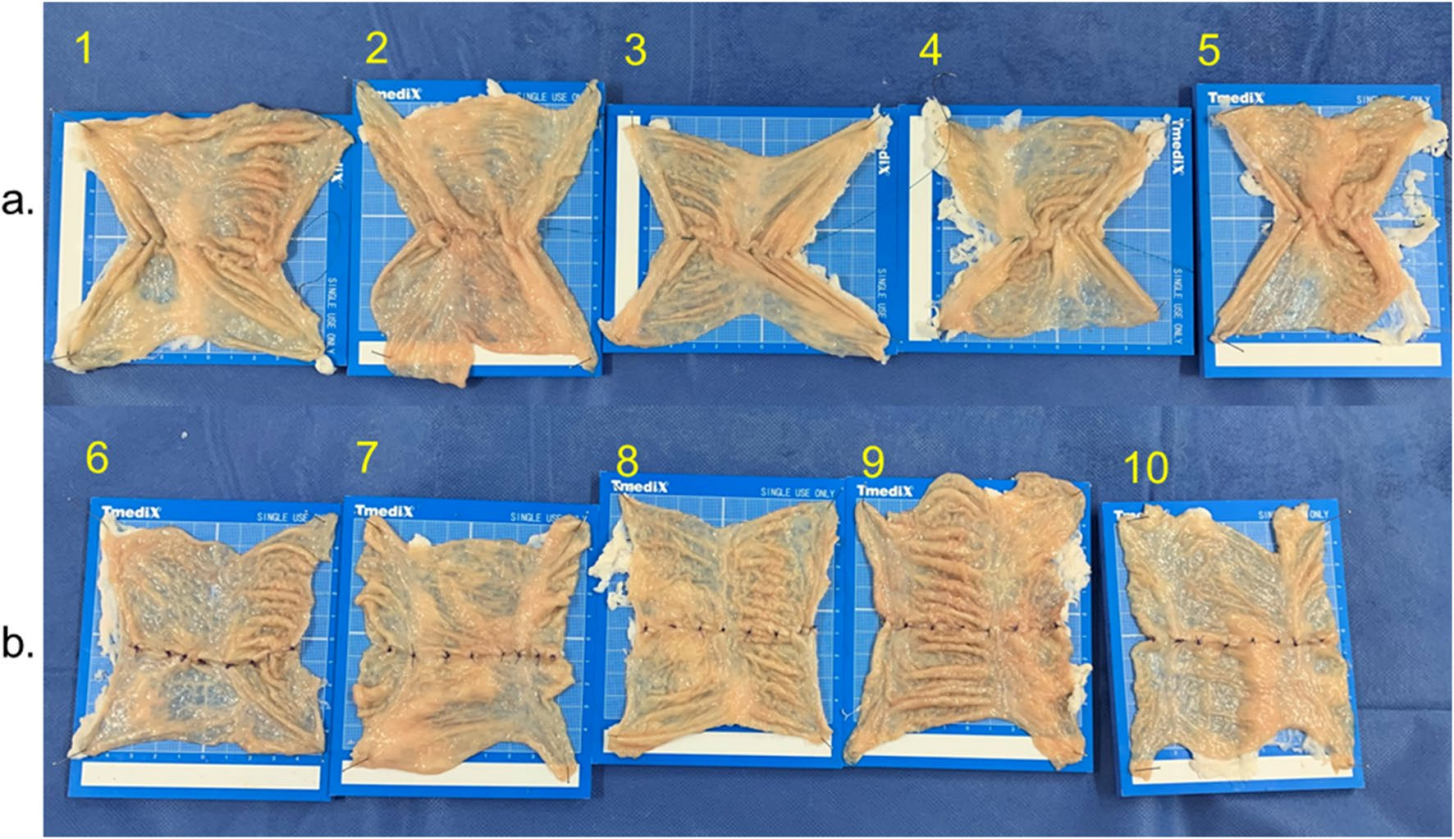
Flat porcine colon models which sutured manually with continuous suturing method and nodule-suturing method. The flat colon models were manually sutured in line by using two types of suturing method. (**a**) Case 1–5: Cases sutured by the continuous suturing method. (**b**) Case 6–10: Cases sutured by the nodule-suturing method. The shortening rate of flat colon models by suturing with the continuous suturing method was greater than that by the nodule suturing method.

In the experiment on the stenosis between two suturing methods, the median shortening rate with the continuous suturing method was significantly higher than that with the nodule-suturing method (48% [range 30–52.6%] vs. 10% [range 0–15%], *p* < 0.001) (Table 2, Fig. 5).

**Table 2:** Outcomes of manual suturing of flat porcine colon model.

Case no.	Suturing method	Thread	Length of flat colon model, cm		Shortening rate, %
			Before suturing	After suturing	
1	Continuous suturing	3-0 V-loc 180^TM^	10.0	7.0	30.0
2	Continuous suturing	3-0 V-loc 180^TM^	10.0	5.2	48.0
3	Continuous suturing	3-0 V-loc 180^TM^	10.0	5.5	45.0
4	Continuous suturing	3-0 V-loc 180^TM^	9.0	4.5	50.0
5	Continuous suturing	3-0 V-loc 180^TM^	9.5	4.5	52.6
6	Nodule suturing	3-0 Biosyn^TM^	10.0	8.5	15.0
7	Nodule suturing	3-0 Biosyn^TM^	10.0	9.5	5.0
8	Nodule suturing	3-0 Biosyn^TM^	9.5	8.5	10.5
9	Nodule suturing	3-0 Biosyn^TM^	9.5	9.5	0.0
10	Nodule suturing	3-0 Biosyn^TM^	10.0	9.0	10.0

## Discussion

In this ex vivo study, we demonstrated that EHS on rectal anastomosis was technically feasible with the acceptable procedure time. Particularly in the nodule-suturing method, no stenoses were seen, and the suturing speed was accelerated depending on the learning effect.

Regarding the reinforcement of the anastomotic site to reduce the possibility of postoperative leakage, several methods, e.g., trans-anal reinforcing anastomotic sutures using a rigid endoscope^4^ and single-stapling with transabdominal reinforcing suture^9,10^, have been reported. However, there remain cases in which anastomosis is difficult to approach either transabdominally or transanally with conventional techniques. Laparoscopic approach will be challenging at the dorsal side of the rectum, particularly in cases with the narrow pelvis. In contrast, difficulty in the transanal suturing with a rigid scope will be increased as the anastomotic site becomes far from the anus. This technique has the flexibility in the suturing area both circumferentially and longitudinally. Therefore, it may be more desirable for reinforcing anastomosis, particularly in the above-mentioned cases.

Another endoscopic technique such as clipping, the most popular mucosal closure method, may work similarly to this technique. However, the previous ex vivo study reported that the strength of the tissue apposition was greater in suturing than in clipping^11,14^. It is expected that EHS provides a comparable strength to surgical suturing because the methodology and materials used in EHS are similar to in surgery.

As an unfavorable event, continuous suturing involved stenosis. In contrast, no stenosis was seen in nodule suturing cases. A possible reason may be that the continuous suturing produces a strong tension in a short axis circumferentially enough to narrow the lumen, whereas the tension in that axis produced by the nodule suturing remains small. In our experiment on the degree of stenosis in each suturing method, the shortening rate was obviously greater in the continuous suturing method than in the nodule suturing method, which implied that the shortening force toward the short axis was stronger in the continuous suturing method than in the nodule-suturing method. Because the stenosis should be avoided to affect the long-term outcome and quality of life^15^, the nodule suturing technique with extra corporeal ligation should be applied. In this study, the procedure time of the nodule suturing became shorter, which suggests that suturing speed will become faster as training and practice are accumulated.

This study has several limitations. First, it was an ex vivo, small-number pilot study using the isolated porcine colon. Consequently, no conclusions can be drawn on whether hemostasis will be adequate in an animate model, and it is uncertain whether the observations on stenosis will also carry over to clinical practice. Second, operators were limited to a single endoscopist and assistant having previous clinical experience of EHS. Third, we did not estimate the sealing strength objectively due to lack of an instrument to measure intraluminal pressure or mechanical strength. Fourth, we have not investigated histological assessments at the sutured anastomoses, which can be done in in vivo models. In vivo experiments would be favorable to directly demonstrate the effectiveness of additional suturing for anastomoses. The results of this ex vivo experiment will be able to accelerate subsequent in vivo experiments as a next step.

In conclusion, we demonstrated that EHS of rectal anastomosis was feasible without stenoses by the nodule-suturing method with extra corporeal ligation in ex vivo porcine models. This concept may be one possible option to reduce the risk of anastomotic leakage after rectal surgery.

## Supplementary Information

Supplementary Video 1.
